# Comparing Complication Rates in Procedures Performed in the Operating Room Versus In-Office: A Cohort Study

**DOI:** 10.1177/15589447261433075

**Published:** 2026-04-20

**Authors:** Jordan T. Carter, Justin Rock, Jason Coffman, Kunj Desai, David Person, Ramesh Srinivasan

**Affiliations:** 1 The University of Texas Health Science Center San Antonio, USA; 2 Hand & Upper Extremity Center of San Antonio, TX, USA

**Keywords:** WALANT, complications, hand, in-office, trigger finger, carpal tunnel

## Abstract

**Background:**

In-office Wide Awake Local Anesthesia No Torniquet (WALANT) is an increasingly popular setting for minor hand procedures. The purpose of this study is to compare complication profiles of common procedures performed in the operating room (OR) versus in the office.

**Methods:**

We performed a retrospective cohort study of patients who underwent trigger finger release, hand/wrist mass excision, open carpal tunnel release, first dorsal compartment release, extensor tendon repair, or needle aponeurotomy between January 1, 2018, and December 31, 2022, at a single private practice location. Patients were stratified by the location of their procedure: in-office versus in the OR. Data collected included standard demographic information, procedure information, and complication profile. Continuous variables were analyzed with means/standard deviations and compared using a Student *t* test. Categorical variables were compared using a χ^2^ test. *P* values less than .05 were considered statistically significant.

**Results:**

We identified 2182 patients meeting the inclusion criteria, 1228 in the in-office WALANT group and 954 in the OR group. Patients in the in-office WALANT group were generally older with more medical comorbidities than patients in the OR group. There were no significant differences in complication rates between the groups. However, smokers or patients with autoimmune diseases had a higher complication rate when their procedure was performed in the office as opposed to the OR.

**Conclusions:**

This study provides valuable information for counseling patients as to the setting of their procedure. While in-office WALANT provides a safe and convenient setting for minor procedures in most patients, others may be at increased risk of complication.

## Introduction

In today’s health care environment, there is an ever-present importance placed on finding effective, cost-efficient ways to care for patients.^1^ As a way to meet these demands, in-office Wide Awake Local Anesthesia No Torniquet (WALANT) has become increasingly popular for shorter duration hand surgery procedures. In-office WALANT has been shown to be cost effective,^2^-^5^ have a positive environmental effect,^6^ and be more convenient for patients compared with traditional procedures performed in the operating room (OR) under general or local anesthesia.^5^

The safety of in-office WALANT procedures has been cited as a concern for widespread adoption.^7^ Recent studies have shown that these procedures are safe and effective in select patients.^6,8^ However, few studies have compared in-office WALANT procedures with similar procedures performed in the OR to compare the safety profiles of each.

The purpose of this study was to compare complication rates of in-office WALANT procedures to similar procedures performed in the OR. We hypothesized that the groups would have similar complication profiles.

## Methods

A retrospective chart review approved by the institutional review board at our institution was undertaken for patients undergoing a hand procedure in the OR or in the office at a single private practice hand clinic between January 1, 2018, and December 31, 2022. All procedures were performed by 4 fellowship-trained hand surgeons.

A manual chart review was undertaken to identify patients undergoing isolated trigger finger release, hand/wrist mass excision, open carpal tunnel release, first dorsal compartment release, extensor tendon repair, or needle aponeurotomy within the time period. Patients were excluded if they underwent endoscopic carpal tunnel release, forearm mass excision (not hand/wrist), underwent multiple procedures, or if they did not present to their first postoperative appointment. Data collected included standard demographic information, procedure performed, setting procedure was performed in, intra procedure complications, and post procedure complications.

Adverse events were defined as infection; defined as either superficial requiring only oral antibiotics, or deep requiring formal irrigation and debridement, recurrence of the condition, or incomplete release.

The in-office technique used by all surgeons as well as the in-office WALANT patients included in this study were previously described by Coffman et al.^6^ Cases performed in the OR were performed under standard sterile technique using a chlorhexidine prep and preoperative Ancef. We do not typically discharge patients on prophylactic oral antibiotics in either group.

Continuous variables were analyzed with means/standard deviations and compared using a Student *t* test. Categorical variables were compared using a χ^2^ test. *P* values less than .05 were considered statistically significant. This articles adheres to STROBE guidelines.

## Results

We identified 2182 patients meeting the inclusion criteria, 1228 in the in-office WALANT group and 954 in the OR group. Demographic information is summarized in Table 1. Patients undergoing in-office WALANT were generally older and had more preoperative comorbid conditions than those whose procedures were performed in the OR. Procedure information can be found in Table 2. There were significant differences in the distribution of procedures performed in the OR versus in-office WALANT.

**Table 1.** Preoperative Demographic Information.

Demographic	WALANT (n = 1228)	OR (n = 954)	*P* value
Age (mean, range)	61 (14.2)	56 (17.3)	<.001
Gender (F)	682 (55.5%)	594 (62.3%)	<.001
PMH			
Diabetes	259 (21.1%)	164 (17.2%)	.022
Hypertension	358 (29.2%)	235 (24.6%)	.018
Autoimmune disease	30 (2.4%)	43 (4.5%)	.007
Smoking	42 (3.4%)	49 (5.1%)	.06

*Note.* WALANT = procedures performed in office wide awake, local anesthesia, no torniquet; OR = procedures performed in the operating room; PMH = past medical history.

**Table 2.** Procedures Performed.

	WALANT (n = 1228)	OR (n = 954)	*P* value
Procedure (%)			<.001
Trigger finger release	962 (78.3%)	271 (28.4%)	
Mass excision	137 (11.2%)	274 (28.7%)	
Carpal tunnel release	33 (2.7%)	290 (30.4%)	
First dorsal compartment release	24 (2.0%)	58 (6.1%)	
Extensor tendon repair	23 (1.9%)	43 (4.5%)	
Foreign body removal	16 (1.3%)	24 (2.5%)	
Needle aponeurotomy	7 (0.5%)	0 (0%)	

*Note.* WALANT = procedures performed in office wide awake, local anesthesia, no torniquet; OR = procedures performed in the operating room.

There was a total of 34 complications (2.77%) identified in the WALANT group versus 41 (4.3%) in the OR group, *P* = .051. Complication rates by procedure can be found in Table 3. There were no significant differences when stratified by procedure. Complication rate by medical comorbidity can be found in Table 4. Patients with autoimmune conditions or who smoked were more likely to have a complication when their procedure was performed in-office. Patients with hypertension had higher complication rates during OR procedures.

**Table 3.** Complications Rates by Procedure.

	Trigger finger release		Mass excision		Carpal tunnel release		First dorsal compartment release		Extensor tendon repair		Foreign body removal		Needle aponeurotomy	
	WALANT	OR	WALANT	OR	WALANT	OR	WALANT	OR	WALANT	OR	WALANT	OR	WALANT	OR
Complications (rate)	26 (2.7%)	13 (4.8%)	5 (3.7%)	14 (5.1%)	0	10 (3.5%)	2 (8.3%)	1 (1.7%)	1 (4.3%)	3 (7.0%)	0	0	0	0
Superficial infection	16 (61.5%)	4 (30.8%)	0	6 (42.8%)	0	9 (90%)	1 (50%)	1 (100%)	1 (100%)	1 (33.3%)	0	0	0	0
Deep infection	3 (11.5%)	3 (23.1%)	0	1 (7.2%)	0	0	0	0	0	1 (33.3%)	0	0	0	0
Recurrence/incomplete release	7 (27%)	6 (46.1%)	5 (100%)	7 (50%)	0	1 (10%)	1 (50%)	0	0	1 (33.3%)	0	0	0	0
*P* value (complications)	.08		.51		.61		.14		.67		0		0	

*Note.* WALANT = procedures performed in office wide awake, local anesthesia, no torniquet; OR = procedures performed in the operating room.

**Table 4.** Complications by Premorbid Condition.

Comorbidity (rate)	Total	WALANT	OR	*P* value
Diabetes	17 (4.0%)	11 (4.2%)	6 (3.6%)	.76
Hypertension	17 (2.9%)	6 (1.7%)	11 (4.68%)	.04
Autoimmune	9 (12.3%	7 (23.3%)	2 (4.65%	.02
Smoking	13 (14.2%)	10 (23.8%)	3 (6.12%)	.03

*Note.* WALANT = procedures performed in office wide awake, local anesthesia, no torniquet; OR = procedures performed in the operating room.

## Discussion

We showed significant differences in the patients and procedures selected for in-office WALANT versus in the OR, but no difference in overall complication profile. Patients undergoing their procedure in the office were older and had more premorbid complications than those undergoing their procedure in the OR yet did not have increased complications. We did show an increased complication rate in patients who smoked or had an autoimmune disease who underwent in-office WALANT procedures compared with procedures performed in the OR.

Patients chosen for in-office WALANT were older on average and had more medical comorbidities than those who had their procedure performed in the OR. This could be due to an aversion to general anesthesia for this population. Older patients and patients with diabetes have been shown to have increased risk of postoperative complications when undergoing orthopedic or general surgeries.^9^ While in-office WALANT may come with its own risks including vasovagal syncope related to the patient being awake for the anesthesia and procedure,^2^ it is able to negate the risks of circulatory collapse or prolonged intubation quoted in studies involving general anesthesia.^9^ This is particularly important in patients with certain pathologies for whom preoperative anesthesia clearance would be an issue or those unwilling to accept the risks of general anesthesia.

There were significant differences in the types of procedures performed in the office versus in the OR with trigger finger release the most common procedure via in-office WALANT and carpal tunnel release in the OR. This could be due to several potential factors including surgeon comfort with the procedure, patient decision, or OR availability. Patient anxiety related to being completely awake for their procedure has traditionally been an issue signing patients up for WALANT surgery.^7^ However, with appropriate patient selection and counseling, recent studies have shown that perioperative anxiety levels were less in patients undergoing a WALANT procedure.^2,7,10,11^ Note that this study’s time period occurred during the COVID-19 pandemic, which restricted the use of ORs and may have influenced the decision to perform procedures in the office versus in the OR. Despite these challenges and adversaries, WALANT was a viable way to keep a hand practice running despite limited resources.^1,12^ In addition, this increased utilization of WALANT during the pandemic^2,13^ may have influenced utilization afterward and increased physician confidence to perform certain procedures in the office.

Trigger finger release is one of the most common procedures performed via in-office WALANT.^2,7,8,11^ Similarly, this was the most common office procedure in our study with a complication rate of 2.7% in the in-office group and 4.8% in the OR group, with superficial infection being the most common complication in the in-office group and recurrence the most common complication in the OR group. Kazmers et al performed a database study comparing trigger finger release performed in the OR versus in the procedure room. Similarly, they found no increase in major medical complications between groups, though they were unable to analyze recurrence rates such as this study.^8^ In addition, the use of perioperative anticoagulation or antiplatelet medications was not associated with an increased risk for revision surgery, further expanding the safety profile of in-office WALANT procedures.^14^

While prior studies have failed to find increased complication rates in patients undergoing in-office WALANT procedures, we were able to identify 2 groups of patients with increased risk of complications, patients with autoimmune disease and smokers. Coffman et al^6^ first demonstrated increased complications in these populations in relation to other patients undergoing in-office WALANT procedures. We further demonstrated that these patients also had increased complications compared with patients with autoimmune disease or a history of smoking undergoing similar procedures in the OR. While both autoimmune disease and smoking have been associated with increased risk of wound complications in hand surgery,^15,16^ we must carefully weigh the risk of increased complications in WALANT surgery versus the increased anesthetic risks seen in this population.^16,17^ This study provides valuable information for counseling patients regarding the risks of in-office WALANT procedures versus those performed in the OR.

This study is not without limitations. Due to the retrospective nature of the study, a selection bias may be present in the way patients were allocated to the OR versus in-office WALANT groups based on the shared decision-making process between them and the physician. Second, only 1 physician in our group regularly performs open carpal tunnel release in the OR while the other 3 typically perform endoscopic carpal tunnel release in the OR and open via WALANT, potentially skewing patients away from in-office open carpal tunnel release. Third, while we were able to capture complications in patients presenting to our clinic, we may have missed others who presented to outside practices or emergency rooms artificially lowering our complication rates. In addition, as mentioned above, a portion of the study did occur during the COVID-19 pandemic, which may have influenced the procedures performed or patient selection process due to significant OR constraints. Despite these limitations, we do believe that the results of our study are valid and provide valuable information for counseling patients in regard to choosing a location for hand procedures.

## Conclusions

This study showed no difference in overall complication rates when comparing in-office WALANT procedures versus procedures performed in the OR despite patients in the WALANT group being older and with more medical comorbidities than those undergoing similar procedures in the OR. We identified patients with autoimmune diseases and smokers at increased risk for complication when their procedure was performed in-office rather than in the OR. This is important for counseling patients regarding the setting of their procedure as we must balance the increased rate of wound complications seen in these patients with the increased rate of anesthetic complications seen in both populations.
